# Endothelial-to-mesenchymal transition in atherosclerosis: mechanisms, therapeutic targets, and future perspectives

**DOI:** 10.3389/fcell.2026.1850232

**Published:** 2026-06-30

**Authors:** Xiaoshan Cui, Yuanyuan Chen, Hailang Luo, Hongzheng Li, Zikai Yu, Hao Guo

**Affiliations:** 1 National Clinical Research Center for Chinese Medicine Cardiology, Xiyuan Hospital, China Academy of Chinese Medical Sciences, Beijing, China; 2 China Academy of Chinese Medical Sciences, Beijing, China; 3 Institute of Basic Medicine, Xiyuan Hospital, China Academy of Chinese Medical Sciences, Beijing, China; 4 Guang’anmen Hospital, China Academy of Chinese Medical Sciences, Beijing, China; 5 Faculty of Medicine, University of British Columbia, Vancouver, BC, Canada; 6 Safety Evaluation Laboratory, Xiyuan Hospital, China Academy of Chinese Medical Sciences, Beijing, China

**Keywords:** atherosclerosis, endothelial plasticity, endothelial-to-mesenchymal transition, epigenetic regulation, metabolic reprogramming, single-cell RNA sequencing, spatial transcriptomics, targeted therapy

## Abstract

Endothelial-to-mesenchymal transition (EndMT) is an endothelial plasticity program that contributes to vascular remodeling, inflammation, extracellular matrix remodeling, calcification, and plaque instability in atherosclerosis. Recent lineage-tracing, single-cell RNA sequencing, and spatial transcriptomic studies have revealed that EndMT is not a uniform or irreversible process, but rather a spectrum of partial, intermediate, and advanced endothelial transition states with stage- and region-specific effects. During atherosclerosis, EndMT may participate in lesion initiation, plaque progression, fibrous cap remodeling, and advanced plaque vulnerability. Mechanistically, EndMT is regulated by interconnected metabolic, signaling, transcriptional, epigenetic, and biomechanical pathways, including TGF-β/SMAD, FGF/FGFR1, BMP, Notch, Wnt/β-catenin, KLF2/KLF4, glycolysis–lactate–lactylation, fatty acid oxidation, HDACs, non-coding RNAs, and extracellular vesicle-mediated communication. Therapeutically, EndMT-targeted strategies should aim to prevent or reverse early maladaptive EndMT while selectively restraining sustained inflammatory, osteogenic, fibroblast-like, or matrix-degrading EndMT states. However, clinical translation remains limited by marker nonspecificity, vascular-bed heterogeneity, disease-stage dependence, inadequate modeling of human plaque rupture, and the lack of validated biomarkers. Future integration of lineage tracing, single-cell and spatial multi-omics, human-relevant models, plaque-risk stratification, and targeted delivery systems may enable precise modulation of EndMT to slow atherosclerosis progression and improve plaque stability.

## 1 Introduction

Atherosclerosis is a chronic, progressive vascular disease characterized by lipid deposition in the intima, infiltration of inflammatory cells, and abnormal proliferation and migration of smooth muscle cells, leading to the formation of atherosclerotic plaques. These plaques cause narrowing or hardening of the arterial lumen (Jaffer et al., 2025; Xie et al., 2025; Kaw et al., 2023). As the disease progresses, the arterial wall thickens, its elasticity decreases, and the risk of plaque rupture increases, potentially leading to life-threatening complications such as myocardial infarction, stroke, peripheral vascular disease and other life-threatening vascular complications (Björkegren and Lusis, 2022; Xu et al., 2022). Endothelial cells (ECs), as the innermost barrier of the vascular wall, are essential for maintaining vascular homeostasis by regulating vascular tone, permeability, leukocyte adhesion, thrombosis, inflammation, and responses to hemodynamic forces (Souilhol et al., 2018; Li X. R. et al., 2019).

Endothelial-to-mesenchymal transition (EndMT) is a dynamic endothelial plasticity program in which ECs progressively lose endothelial characteristics and acquire mesenchymal-like features under specific physiological or pathological stimuli (Li et al., 2018). This process is characterized by reduced endothelial markers, such as CD31, VE-cadherin, vWF, eNOS, and ZO-1/2, together with increased mesenchymal or fibroblast-like markers, including α-SMA, vimentin, FSP1/S100A4, COL1A1, fibronectin, and ZEB1/2 (Kovacic et al., 2019; Piera-Velazquez and Jimenez, 2019). Functionally, EndMT-like cells may display enhanced migratory, invasive, extracellular matrix-producing, and matrix-remodeling capacities. Through these changes, EndMT may contribute to vascular remodeling, inflammatory microenvironment formation, fibrosis, calcification, endothelial barrier disruption, and plaque instability in atherosclerosis.

Although EndMT shares several core regulatory features with classical epithelial-to-mesenchymal transition (EMT), including activation of TGF-β/SMAD signaling and transcription factors such as SNAI, ZEB, and TWIST, it has distinct endothelial-specific characteristics. Unlike EMT, EndMT originates from ECs and involves the loss of endothelial barrier integrity, nitric oxide production, anti-thrombotic properties, intercellular junctions, and shear-stress sensing (Dong et al., 2023). Endothelial-specific programs, including KLF2/KLF4, ERK5, FGFR1-dependent inhibition of TGF-β signaling, VE-cadherin/eNOS-associated endothelial identity, and mechanotransduction pathways, confer unique regulatory features to EndMT in the vascular microenvironment. In atherosclerosis, these endothelial-specific programs are further shaped by disturbed flow, oxidized lipids, oxidative stress, hyperglycemia, inflammatory cytokines, immune-cell crosstalk, and plaque matrix remodeling (Bleckwehl et al., 2024; Adelus et al., 2024).

Recent studies have shown that EndMT in atherosclerosis is regulated by interconnected metabolic, signaling, transcriptional, epigenetic, and biomechanical mechanisms (Zhao et al., 2023; Dong et al., 2024). Importantly, EndMT should not be viewed as a uniform, irreversible, or universally pathogenic process (Tombor et al., 2021). Instead, it may occur as a spectrum of partial, intermediate, and advanced endothelial-transition states with distinct functional consequences at different stages and regions of atherosclerotic plaques (Tardajos Ayllon et al., 2026). Building on these insights, this review summarizes recent advances in EndMT biology in atherosclerosis, with emphasis on disease-stage-specific roles, marker and lineage-tracing evidence, metabolic and signaling regulation, endothelial heterogeneity, spatial context within plaques, and emerging therapeutic strategies. We further discuss the translational challenges of targeting EndMT, including marker specificity, vascular-bed heterogeneity, timing of intervention, and the need to selectively suppress maladaptive EndMT while preserving endothelial repair and adaptive matrix remodeling.

## 2 Role of EndMT in atherosclerosis

### 2.1 EndMT, endothelial dysfunction, and phenotypic plasticity

**FIGURE 1:**
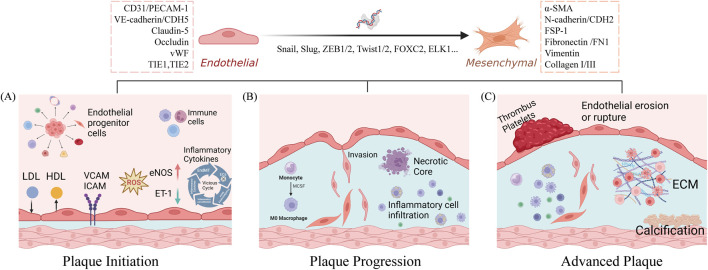
EndMT in atherosclerotic plaque evolution. Transcription factors (Snail, Slug, ZEB, Twist, FOXC2, ELK1) trigger the loss of endothelial markers (CD31, VE-cadherin) and gain of mesenchymal markers (α-SMA, N-cadherin, fibronectin), converting endothelial cells into invasive, matrix-producing cells. EndMT accelerates **(A)** plaque initiation via lipid uptake, ROS production and leukocyte adhesion; **(B)** plaque progression through intimal invasion, necrotic-core growth and inflammation; and **(C)** advanced-plaque instability by ECM deposition, calcification, endothelial rupture and thrombosis. Created with BioRender.com. Abbreviations: LDL, low-density lipoprotein; HDL, high-density lipoprotein; VCAM-1/ICAM-1, adhesion molecules; ROS, reactive oxygen species; eNOS, endothelial NO synthase; ET-1, endothelin-1; ECM, extracellular matrix; EndMT, endothelial-to-mesenchymal transition.

EndMT is a dynamic endothelial plasticity program with physiological roles in embryonic development and tissue repair, whereas its pathological activation contributes to vascular fibrosis, inflammatory microenvironment remodeling, excessive extracellular matrix (ECM) deposition, osteogenic differentiation, and disruption of vascular wall homeostasis (Hall et al., 2024). In atherosclerosis, EndMT is increasingly recognized as an important contributor to lesion initiation, plaque progression, and advanced plaque remodeling. These stage-specific roles of EndMT in atherosclerotic plaque evolution are illustrated in Figure 1.

Endothelial dysfunction may act as an upstream trigger or accompanying event of EndMT; however, these are two related but distinct biological processes. Endothelial dysfunction mainly refers to functional abnormalities of endothelial cells, including reduced nitric oxide production, impaired endothelium-dependent vasodilation, increased permeability, oxidative stress, leukocyte adhesion, and inflammatory activation (Wang and He, 2024). By contrast, EndMT represents a phenotypic transition in which endothelial cells progressively lose endothelial identity and acquire mesenchymal, migratory, inflammatory, and matrix-producing features. Once initiated, EndMT may further aggravate endothelial barrier disruption, inflammatory amplification, and matrix remodeling, thereby linking endothelial injury to atherosclerotic plaque development.

Identification of EndMT in atherosclerosis remains challenging because no single marker is sufficiently specific to define endothelial-derived mesenchymal cells *in vivo* (Evrard et al., 2016). Current practice relies on combined evidence, including reduced endothelial markers, such as CD31, VE-cadherin, vWF, eNOS, and ZO-1, together with increased mesenchymal or fibroblast-like markers, such as α-SMA, vimentin, FSP1/S100A4, COL1A1, fibronectin, SNAI1/2, ZEB1/2, TWIST, and fibroblast activation protein (FAP) (Table 1). However, these mesenchymal markers differ substantially in specificity. α-SMA can also be expressed by vascular smooth muscle cells (VSMCs) and myofibroblasts; COL1A1 and fibronectin are broadly expressed by matrix-producing stromal cells; and FSP1/S100A4 may also appear in inflammatory or fibroblast-like populations (Alencar et al., 2020; Newman et al., 2021). FAP is useful for identifying activated fibroblast-like or matrix-remodeling cells, but FAP expression alone cannot prove endothelial origin.

**TABLE 1:** Common markers used to identify EndMT in atherosclerosis and their limitations.

Marker category	Representative markers	Interpretation	Major limitation
Endothelial markers	CD31, VE-cadherin, vWF, eNOS, ZO-1	Loss indicates reduced endothelial identity	Downregulation may also occur during endothelial dysfunction without EndMT
Mesenchymal markers	α-SMA, vimentin, FSP1/S100A4, COL1A1, fibronectin	Gain suggests mesenchymal-like transition	Not specific to endothelial-derived cells
Transcription factors	SNAI1/2, ZEB1/2, TWIST	Indicate activation of EndMT transcriptional program	Also involved in EMT, fibrosis, and other cell-state transitions
Fibroblast-like markers	FAP, COL1A1, fibronectin	Suggest activated fibroblast-like or matrix-remodeling phenotype	FAP is not specific for EndMT and cannot prove endothelial origin
Lineage evidence	Endothelial-specific lineage tracing/fate mapping	Supports endothelial origin	Mostly available in animal models; depends on promoter specificity and recombination efficiency
Omics evidence	scRNA-seq, spatial transcriptomics	Defines transitional states and plaque localization	Requires validation; transcriptomic similarity alone does not prove lineage origin

Therefore, robust identification of EndMT requires integration of endothelial lineage tracing or fate mapping, endothelial–mesenchymal marker co-expression, single-cell transcriptomic signatures, spatial localization, and, where possible, functional validation. Recent single-cell RNA sequencing studies have revealed substantial endothelial heterogeneity within atherosclerotic plaques, including inflammatory, disturbed-flow-responsive, proliferative, angiogenic, and mesenchymal-like endothelial subsets (Bashore et al., 2024; Traeuble et al., 2024). Spatial transcriptomics further extends these findings by mapping EndMT-associated signatures to specific plaque regions, such as the luminal endothelium, plaque shoulder, fibrous cap, neovessels, necrotic core border, and calcified areas (Bleckwehl et al., 2024; Pauli et al., 2025). However, transcriptomic similarity alone cannot definitively establish endothelial origin. Thus, single-cell and spatial omics should be interpreted together with lineage-based and functional evidence to distinguish true EndMT-derived cells from VSMC-, fibroblast-, or pericyte-derived populations with overlapping mesenchymal signatures.

### 2.2 EndMT in atherosclerosis initiation and plaque progression

During atherosclerosis initiation, disturbed flow, hyperglycemia, oxidative stress, hypoxia, lipid stress, and inflammatory mediators first induce endothelial dysfunction, characterized by impaired endothelium-dependent vasodilation, increased permeability, leukocyte adhesion, and inflammatory activation (Evrard et al., 2017; Zhang et al., 2024). Under persistent stimulation, dysfunctional endothelial cells may further enter a partial EndMT state, thereby amplifying endothelial injury and promoting early lesion formation.

At this early stage, partial EndMT-like changes may enhance endothelial barrier disruption, leukocyte adhesion, lipid entry, and local inflammatory activation, thereby facilitating early atherogenesis. Inflammatory stimulation may further reinforce EndMT, creating a feed-forward loop in which endothelial injury, immune-cell recruitment, and endothelial plasticity amplify one another (Helmke et al., 2019; Chen et al., 2020). Thus, in lesion initiation, EndMT may serve as a mechanistic link between endothelial dysfunction and early plaque-forming events.

As plaques form and progress, endothelial cells undergoing EndMT may acquire mesenchymal-like phenotypes and increased migratory capacity, allowing them to contribute to plaque cellular heterogeneity and matrix remodeling. Endothelial lineage-tracing studies have demonstrated that endothelial-derived fibroblast-like cells are present within atherosclerotic plaques and can express mesenchymal or fibroblast-associated markers (Evrard et al., 2017). This provides important *in vivo* evidence that endothelial cells can contribute to mesenchymal-like cell populations within plaques.

However, the proliferative dynamics of EndMT-derived cells remain incompletely defined. Although lineage tracing supports the endothelial origin of these cells, direct evidence for clonal expansion of EndMT-derived cells in atherosclerosis remains limited. This contrasts with VSMCs, for which lineage-tracing and clonal analyses have shown that a relatively small number of medial VSMCs can expand and generate diverse plaque cell populations (Misra et al., 2018; Kawai et al., 2023). Thus, current evidence suggests that EndMT contributes to plaque remodeling mainly through endothelial phenotypic transition and acquisition of mesenchymal-like features, whereas whether EndMT-derived cells undergo monoclonal or oligoclonal expansion remains unclear.

EndMT-derived cells may also interact with VSMCs, macrophages, and other plaque-resident cells through cytokines, chemokines, ECM components, extracellular vesicles, and inflammatory mediators. Through these interactions, EndMT may contribute to vascular wall remodeling, immune-cell recruitment, ECM remodeling, and plaque progression (Helmke et al., 2019; Pauli et al., 2025). Whether these effects are primarily reparative or maladaptive likely depends on disease stage, local inflammatory burden, and the persistence of the EndMT program.

### 2.3 Contribution of EndMT-derived cells to fibrous cap remodeling

Fibrous cap integrity is primarily maintained by VSMC-derived collagen-rich ECM. VSMCs remain the principal and best-established cellular source of fibrous cap formation and ECM production in atherosclerotic plaques. Through phenotypic switching toward synthetic, fibromyocyte-like, or fibroblast-like states, VSMCs produce collagen, elastin, proteoglycans, fibronectin, and other matrix components that are essential for fibrous cap strength and plaque stabilization (Wirka et al., 2019; Goncalves et al., 2026).

In this context, EndMT-derived cells should be considered modulators rather than dominant structural determinants of the fibrous cap. Under partial or reparative EndMT, endothelial-derived mesenchymal-like cells may acquire fibroblast-like or myofibroblast-like properties and contribute to local ECM deposition, including collagen, fibronectin, and proteoglycans. Such responses may support fibrous cap remodeling or repair, particularly when matrix production predominates over matrix degradation (Newman et al., 2021; Tardajos Ayllon et al., 2026).

Conversely, under persistent inflammatory stimulation, EndMT-like cells may produce cytokines, chemokines, and matrix-degrading enzymes such as matrix metalloproteinases (MMPs). These mediators may promote macrophage recruitment, collagen degradation, ECM disorganization, and cap thinning (Evrard et al., 2016). Therefore, the contribution of EndMT to fibrous cap remodeling is likely bidirectional. Controlled or partial EndMT may support adaptive matrix remodeling and cap repair, whereas sustained inflammatory EndMT may weaken the fibrous cap by enhancing inflammatory signaling, MMP activity, and collagen degradation. Accordingly, VSMCs mainly determine the structural framework of the fibrous cap, while EndMT-derived cells may influence plaque stability by regulating endothelial barrier integrity, inflammatory signaling, and the balance between matrix synthesis and matrix degradation.

### 2.4 EndMT in advanced plaque instability and rupture-prone remodeling

In advanced atherosclerotic plaques, EndMT is associated with several rupture-prone features, including endothelial barrier disruption, inflammatory amplification, MMP-mediated matrix degradation, calcification, and fibrous cap weakening (Yao et al., 2015; Boström et al., 2016). Notably, plaque rupture is not caused by EndMT alone, but rather results from the combined effects of endothelial injury, VSMC dysfunction, macrophage-driven inflammation, ECM degradation, necrotic core expansion, and calcification.

VSMC dysfunction is particularly important because it directly compromises the structural strength of the fibrous cap (Goncalves et al., 2026). Loss of VSMCs, VSMC apoptosis or senescence, reduced collagen synthesis, and maladaptive VSMC phenotypic switching can weaken the cap and increase rupture risk (Kaistha, 2025). Importantly, VSMC defects may also influence the endothelial compartment. Recent evidence suggests that VSMC dysfunction can alter endothelial cell states, activate TGF-β/SMAD-dependent signaling, and induce EndMT, thereby linking VSMC injury to endothelial phenotypic transition and accelerated plaque remodeling (Hamczyk et al., 2024). Thus, VSMC dysfunction may promote plaque vulnerability through both direct structural weakening of the fibrous cap and indirect aggravation of endothelial maladaptation and EndMT.

Several EndMT-related mechanisms may contribute to advanced plaque instability. Activation of bone morphogenetic protein (BMP) signaling can induce endothelial phenotypic transition and promote osteogenic or calcification-associated remodeling (Malhotra et al., 2015). EndMT-like cells may also secrete pro-inflammatory mediators that amplify macrophage recruitment and local inflammatory activation (Lecce et al., 2021; Singh B. et al., 2024). In addition, EndMT has been linked to increased MMP-9 activity, and inhibition of MMP-9 can attenuate EndMT-associated pathological remodeling in experimental models (He et al., 2024). These findings suggest that EndMT-related inflammatory, osteogenic, and matrix-degrading programs may interact with VSMC phenotypic switching and macrophage activation to shape the balance between plaque stabilization and rupture-prone remodeling.

Nevertheless, current evidence mainly supports an association between EndMT-derived or EndMT-like cells and unstable plaque features. Direct proof that EndMT alone is sufficient to cause spontaneous plaque rupture remains limited. Therefore, EndMT should be interpreted as one component of a broader rupture-prone plaque microenvironment rather than as an isolated cause of plaque rupture.

### 2.5 Context-dependent effects of EndMT and current evidence limitations

The role of EndMT in atherosclerosis is context dependent and stage specific. Although maladaptive EndMT has been linked to endothelial barrier disruption, inflammatory amplification, macrophage recruitment, MMP activation, ECM disorganization, calcification, and plaque instability, EndMT should not be viewed as uniformly harmful. Partial and transient EndMT may support endothelial plasticity, wound repair, ECM deposition, and fibrous cap remodeling, thereby contributing to reparative matrix production and plaque stabilization (Newman et al., 2021; Tardajos Ayllon et al., 2026).

In contrast, sustained or inflammatory EndMT may impair endothelial integrity, promote leukocyte recruitment, increase cytokine and chemokine production, enhance matrix degradation, and facilitate calcification, ultimately promoting plaque vulnerability (Sánchez-Duffhues et al., 2019; Hamczyk et al., 2024). Thus, the biological consequence of EndMT depends not only on mesenchymal marker expression, but also on disease stage, plaque microenvironment, spatial localization, degree of endothelial transition, and reversibility of the EndMT state. Based on current evidence, EndMT is better understood as a spectrum of endothelial plasticity states rather than a binary or universally pathogenic process (Goncalves et al., 2026; Tardajos Ayllon et al., 2026).

Taken together, available *in vivo* evidence supports a role for EndMT in atherosclerotic lesion initiation, plaque progression, fibrous cap remodeling, calcification, and unstable plaque features. Endothelial lineage-tracing studies provide important evidence that endothelial-derived mesenchymal-like or fibroblast-like cells are present within plaques (Evrard et al., 2017). Genetic and pharmacological studies targeting EndMT-related pathways, including TGF-β/SMAD, BMP signaling, MMP-associated remodeling, mechanotransduction pathways, and epigenetic regulators, further support the involvement of EndMT in plaque inflammation, matrix remodeling, calcification, and vulnerability.

However, several limitations remain. Many studies still rely on marker co-expression, which cannot definitively establish endothelial origin. Lineage tracing provides stronger evidence but is mostly restricted to animal models and may be influenced by promoter specificity, recombination efficiency, labeling timing, and disease-model context. Moreover, current experimental models rarely reproduce spontaneous human plaque rupture, limiting direct translation from EndMT-associated plaque features to rupture causality. Future studies combining endothelial-specific lineage tracing, fate mapping, genetic barcoding, single-cell RNA sequencing, spatial transcriptomics, and functional validation are needed to define the precise contribution of EndMT to each stage of atherosclerosis and to distinguish maladaptive EndMT from potentially reparative endothelial plasticity (Pauli et al., 2025; Goncalves et al., 2026).

## 3 Regulatory mechanisms of EndMT in atherosclerosis

**FIGURE 2:**
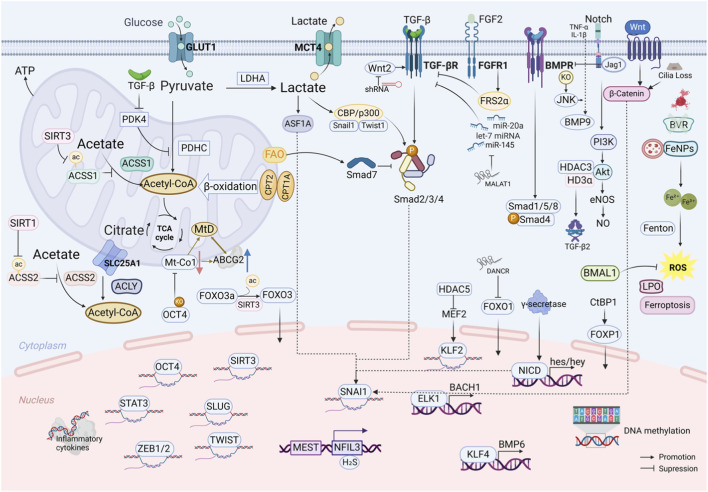
Metabolic, signalling and epigenetic crosstalk driving EndMT in atherosclerosis. Glycolysis/lactate export (GLUT1, LDHA, MCT4), β-oxidation (CPT1), and the acetate-citrate shuttle (ACSS1/2, ACLY) raise acetyl-CoA and histone acetylation, opposed by SIRT1/3. Convergent TGF-β/Smad, BMP, FGFR1, Notch/β-catenin, PI3K-Akt-eNOS and JNK pathways—potentiated by ROS-ferroptosis—promote EndMT, whereas shear-stress sensors KLF2/FOXO1 dampen it. HDACs, DNA methylation, lncRNAs (MALAT1, DANCR) and miRNAs (let-7, miR-145) tune a core transcriptional module (Snail, Slug, ZEB, TWIST, STAT3, ELK1) that reprograms endothelial identity. Solid arrows = activation; blunt lines = inhibition. Created with BioRender.com. Abbreviations: ACLY, ATP-citrate lyase; ACSS, acyl-CoA synthetase short-chain; CPT1, carnitine palmitoyl-transferase-1; FAO, fatty-acid oxidation; GLUT1, glucose transporter-1; HDAC, histone deacetylase; LDHA, lactate dehydrogenase A; LPO, lipid peroxidation; ROS, reactive oxygen species.

EndMT in atherosclerosis is shaped by converging developmental, inflammatory, metabolic and mechanical cues within the plaque microenvironment. These signals are further modulated by endothelial-bed heterogeneity and by crosstalk with smooth muscle cells, macrophages and fibroblasts through oxidized lipids, cytokines and extracellular vesicles, collectively dictating when and where EndMT contributes to barrier disruption, inflammation, neovascularization and fibrous-cap weakening (Figure 2).

### 3.1 Metabolic regulation

#### 3.1.1 Glycolysis and acetyl-CoA metabolism

Metabolic reprogramming is increasingly recognized as an important regulator of endothelial plasticity and EndMT in atherosclerosis. Acetyl-CoA (Ac-CoA) is a central metabolic intermediate involved in the tricarboxylic acid cycle, lipid synthesis, and protein acetylation. Under physiological conditions, citrate generated in the mitochondria is transported into the cytoplasm via the SLC25A1 transporter, where it is cleaved by ATP-citrate lyase (ACLY) to produce Ac-CoA. This process inhibits EndMT and maintains endothelial stability (Narita et al., 2019). However, under pro-EndMT conditions, such as high glucose, inflammatory stimulation, or TGF-β exposure, glycolytic flux is enhanced and pyruvate-derived Ac-CoA production is increased. Pyruvate is converted to Ac-CoA by the pyruvate dehydrogenase complex (PDHC), while acetyl-CoA synthetase short-chain family member 1 (ACSS1) further amplifies glucose-dependent Ac-CoA accumulation, forming a positive feedback loop that promotes EndMT (Liu et al., 2018; Zhu X. L. et al., 2023).

TGF-β can also promote glucose uptake by upregulating GLUT1 and downregulating pyruvate dehydrogenase kinase 4 (PDK4), thereby activating PDHC and promoting Ac-CoA synthesis. Increased Ac-CoA availability facilitates acetylation of ALK5 and SMAD2/3/4, reinforcing TGF-β signaling and driving EndMT (Recatalá and Santoro, 2023). In the cytoplasm, ACSS2 catalyzes acetate-to-Ac-CoA conversion and is regulated by deacetylases such as SIRT1 and SIRT3 (Schwer et al., 2006; Chow et al., 2014). Importantly, inhibition of ACSS2 disrupts this Ac-CoA amplification loop and reduces atherosclerotic plaque formation, suggesting that glycolysis–Ac-CoA coupling is not only a general EndMT mechanism but also a disease-relevant metabolic axis in atherosclerosis (Zhu X. L. et al., 2023). These findings are particularly relevant to diabetes-associated atherosclerosis, where persistent hyperglycemia may promote EndMT through glycolytic activation, oxidative stress, lactate accumulation, and TGF-β-dependent signaling.

#### 3.1.2 Lactate production and lactylation

Lactate, another major product of enhanced glycolysis, is not merely a metabolic by-product but also functions as a signaling molecule that regulates epigenetic modifications and transcriptional programs. Lactate can enhance the interaction between Snail1 and the acetyltransferase CBP/p300 complex, promoting Snail1 lactylation and activating the TGF-β/SMAD2 pathway, thereby inducing EndMT and disrupting endothelial barrier function (Fan et al., 2022). A similar mechanism has been reported for TWIST1, where high lactate levels enhance CBP/p300-mediated lactylation and promote TGF-β-dependent EndMT (Xu et al., 2024). Lactate may also activate the p300/ASF1A complex, increase Snail1 transcription, and establish a lactate–transcription factor–TGF-β positive feedback loop (Dong et al., 2024).

In the context of atherosclerosis, lactate-related EndMT is relevant because plaques are metabolically active, inflamed, and often hypoxic. Such microenvironmental conditions may favor glycolytic reprogramming, lactate accumulation, and endothelial barrier dysfunction. Therefore, maintaining lactate homeostasis or targeting lactate-related enzymes, such as lactate dehydrogenase A (LDHA), may represent a potential strategy to limit pathological EndMT and slow atherosclerotic progression. However, direct *in vivo* evidence linking lactate-lactylation pathways to EndMT in atherosclerotic plaques remains relatively limited, and further plaque-specific validation is needed.

#### 3.1.3 Fatty acid oxidation

Fatty acid oxidation (FAO) is important for maintaining endothelial energy balance and functional homeostasis. Compared with glycolysis, FAO generally exerts a protective role in endothelial cells, and disruption of FAO may facilitate endothelial dysfunction and pro-EndMT transition. Deletion of carnitine palmitoyltransferase 1A (CPT1A) impairs FAO and nucleotide synthesis, thereby affecting endothelial proliferation (Schoors et al., 2015). CPT2 deficiency reduces mitochondrial Ac-CoA production, suppresses SMAD7 expression, enhances TGF-β signaling, and promotes EndMT with increased vascular permeability (Xiong et al., 2018). FAO is also linked to endothelial senescence and inflammatory activation. Maintaining FAO homeostasis helps preserve endothelial function, prevents ECs from entering a pro-EndMT state, delays endothelial aging, and exerts anti-inflammatory and anti-atherosclerotic effects (Guo Y. T. et al., 2024; Pulipaka et al., 2024). From an atherosclerosis-specific perspective, FAO disruption may be particularly relevant in metabolically stressed plaques and diabetic vascular disease, where endothelial mitochondrial dysfunction, oxidative stress, and inflammatory signaling coexist. Thus, FAO-related pathways may influence EndMT both by regulating energy metabolism and by modulating TGF-β/SMAD-dependent endothelial identity loss.

#### 3.1.4 Iron homeostasis and ferroptosis-related stress

Iron homeostasis is closely linked to endothelial redox balance and EndMT regulation. Iron overload can generate reactive oxygen species (ROS) through the Fenton reaction, leading to oxidative stress and activation of EndMT-related transcription factors such as SNAI1/2 (Fang et al., 2023). Deficiency of biliverdin reductase (BVR), which disturbs heme degradation and promotes free iron accumulation, increases the expression of mesenchymal markers, including TWIST1, FAP, and FN1, thereby promoting EndMT (Klóska et al., 2019). Iron oxide nanoparticles may also release Fe^2+^/Fe^3+^ in lysosomes, activate oxidative stress responses, and induce EndMT (Wen et al., 2019). In addition, CXCL8/IL-8 enhances lipid peroxidation, aggravates ferroptosis, and triggers TGF-β2-induced EndMT (Ji et al., 2023). Angiotensin II can further induce EndMT and impair endothelial function by promoting iron overload and Frataxin/SIRT3 degradation (Pulipaka et al., 2024). In atherosclerosis, iron-driven oxidative stress and ferroptosis are highly relevant because plaques contain inflammatory cells, oxidized lipids, and redox-active microenvironments. Therefore, iron overload may contribute to EndMT indirectly by amplifying oxidative injury, endothelial dysfunction, and inflammatory remodeling. Nevertheless, more *in vivo* studies are needed to determine whether iron-dependent EndMT directly contributes to plaque progression, calcification, or instability.

#### 3.1.5 Diabetes-associated metabolic stress

Diabetes creates a permissive vascular milieu for EndMT through persistent hyperglycemia, advanced glycation end-product accumulation, oxidative stress, mitochondrial dysfunction, chronic low-grade inflammation, and altered extracellular vesicle signaling (Khan et al., 2026). High glucose may enhance glycolytic flux and lactate production, activate TGF-β/SMAD signaling, increase ROS generation, and induce epigenetic remodeling, thereby promoting endothelial phenotypic plasticity. In diabetic atherosclerosis, these convergent metabolic and inflammatory cues may drive partial or sustained EndMT, contributing to endothelial barrier disruption, leukocyte adhesion, intimal remodeling, extracellular matrix deposition, and vascular calcification (Tsai et al., 2021; Chang et al., 2022).

In addition, diabetes may amplify EndMT through non-coding RNAs and extracellular vesicles released by metabolically stressed cells (Cao et al., 2021; Wu et al., 2023). Extracellular vesicles derived from high-glucose-conditioned or diabetic cells may deliver pro-fibrotic, pro-inflammatory, or metabolism-associated cargos to endothelial cells and activate TGF-β/SMAD-dependent EndMT programs (Vuong et al., 2024). Accordingly, diabetes-associated EndMT may represent a key mechanistic bridge between metabolic disease, accelerated atherosclerosis, and plaque remodeling.

### 3.2 Signal pathway regulation

#### 3.2.1 TGF-β signaling pathway

The transforming growth factor-β (TGF-β) signaling pathway is one of the central regulators of EndMT, but its role in atherosclerosis is highly cell type- and context-dependent. In endothelial cells, activation of TGF-β signaling can promote endothelial inflammation, barrier disruption, and EndMT-like phenotypic transition, thereby contributing to plaque inflammation and vascular remodeling (von Gise and Pu, 2012; Gonzalez and Medici, 2014; Chen et al., 2019). In contrast, TGF-β signaling in vascular smooth muscle cells (VSMCs) may promote a contractile phenotype and support fibrous cap stability under certain conditions (Chen et al., 2016). This cell-specific difference is important for therapeutic translation, because broad inhibition of TGF-β signaling may suppress pathological endothelial transition but could also interfere with protective VSMC functions.

Mechanistically, TGF-β induces EndMT through both SMAD-dependent and SMAD-independent pathways. In the canonical pathway, TGF-β binds to TGF-β receptor II (TβRII) and activates TGF-β receptor I (TβRI/ALK5), leading to phosphorylation of SMAD2/3 and, in some contexts, SMAD1/5. Activated SMAD complexes associate with SMAD4 and translocate into the nucleus, where they cooperate with transcription factors such as SNAI, SLUG, TWIST, ZEB1/2, and FOXC2 to repress endothelial junctional genes and activate mesenchymal gene programs. This process is accompanied by reduced expression of endothelial markers such as VE-cadherin and increased expression of mesenchymal markers such as α-SMA and N-cadherin (Derynck and Zhang, 2003; Cooley et al., 2014). In parallel, non-canonical TGF-β signaling pathways, including TAK1/p38 MAPK, PKC-δ/c-Abl/GSK3β, PI3K/Akt/mTOR, Rho-GTPases/ALK2, and NF-κB, regulate cytoskeletal remodeling, migration, inflammatory responses, and fibrotic matrix production, thereby reinforcing EndMT and plaque remodeling (Sakabe et al., 2008; Li and Jimenez, 2011; Medici et al., 2011; Lee et al., 2017).

Importantly, TGF-β signaling should not be viewed only as a general inducer of EndMT, because its activity is dynamically shaped by the atherosclerotic plaque microenvironment, including inflammatory cytokines, disturbed flow, oxidative stress, metabolic stress, and endothelial-protective pathways. In endothelial cells, excessive or sustained TGF-β activation promotes loss of endothelial identity, inflammatory remodeling, and mesenchymal gene expression. Therefore, therapeutic targeting of TGF-β signaling should consider disease stage, vascular cell type, and potential effects on fibrous cap stability.

#### 3.2.2 FGF/FGFR1 signaling pathway

Compared with pro-EndMT pathways, endogenous anti-EndMT mechanisms have received less attention. Among them, the fibroblast growth factor (FGF)/FGFR1 pathway represents one of the best-characterized protective signaling axes in endothelial cells. In ECs, FGF2 and FGFR1 signaling suppress TGF-β activation, reduce EndMT, and support endothelial proliferation and homeostasis (Xiao and Dudley, 2017). In contrast, FGF signaling in VSMCs may have different effects and may contribute to VSMC proliferation or lesion remodeling depending on disease context (Chen et al., 2016).

FGF binding to FGFR1 recruits FGFR substrate 2α (FRS2α), which inhibits TβRI activity and thereby suppresses TGF-β-driven EndMT (Alvandi and Bischoff, 2021). EC-specific FGFR1 knockdown activates TGF-β signaling and induces EndMT (Chen et al., 2014). Chen et al. further showed that disruption of endothelial FRS2α exacerbates atherosclerotic lesions in mice, supporting the *in vivo* relevance of this pathway (Chen et al., 2015). FGF-mediated EndMT suppression is also linked to let-7 miRNA and miR-20a, which target TGF-β ligands, receptors, and downstream signaling components (Chen et al., 2012; Correia et al., 2016). In addition, Epsins bind ubiquitinated FGFR1 through their UIM domain, induce FGFR1 endocytosis and degradation, enhance TGF-β signaling, and trigger EndMT (Dong et al., 2023). Together, these findings identify the endothelial FGFR1–TGF-β antagonistic axis as a disease-specific mechanism linking endothelial identity loss to atherosclerotic remodeling. Therapeutic strategies that preserve endothelial FGFR1 signaling may therefore restrain maladaptive EndMT while maintaining endothelial repair.

#### 3.2.3 BMP signaling pathway

Bone morphogenetic proteins (BMPs), members of the TGF-β superfamily, signal through BMPR1A, BMPR1B, and BMPR2 to activate SMAD1/5/8 and regulate target gene expression (Katagiri and Watabe, 2016; Lee et al., 2023). BMP signaling contributes to EndMT in several vascular contexts and is particularly relevant to calcification and advanced plaque remodeling. BMPR2 deficiency disrupts the BMPR2/JNK axis in human aortic endothelial cells and reduces resistance to BMP9-mediated EndMT induced by inflammatory cytokines such as TNF-α and IL-1β (Sánchez-Duffhues et al., 2019). Deletion of Krev Interaction Trapped Protein 1 (KRIT1) activates the MEKK3/MEK5/ERK5/MEF2 pathway, induces KLF4 and BMP6 expression, and promotes EndMT (Cuttano et al., 2016; Shoemaker et al., 2020). Disease-specific evidence also links BMP-related EndMT to plaque progression and instability. BMAL1 suppresses ROS-induced EndMT and slows atherosclerotic plaque progression through BMP signaling, suggesting that BMP-related EndMT is closely associated with oxidative stress-driven endothelial remodeling and plaque vulnerability (Zhu et al., 2018). In advanced plaques, BMP-driven EndMT may also contribute to osteogenic remodeling and calcification, thereby connecting endothelial plasticity with plaque stiffness and rupture-prone phenotypes.

#### 3.2.4 Notch signaling pathway

The Notch pathway is activated by ligands such as Delta-like proteins (DLL1/3/4) and Jagged proteins (Jag1/2). Ligand binding triggers γ-secretase-mediated cleavage and release of the Notch intracellular domain (NICD), which translocates into the nucleus and regulates target genes such as HES and HEY (Reichman et al., 2016). Notch signaling can promote EndMT through interactions with TGF-β signaling, inflammatory mediators, and matrix-remodeling pathways (MacGrogan et al., 2016; Lin Q. Q. et al., 2018). However, Notch effects are context dependent, as Jagged1 has also been reported to interfere with BMP-mediated EndMT in certain settings. In atherosclerotic lesions, Notch signaling has been associated with endothelial activation, MMP activity, and plaque remodeling. Notch signaling pathway promotes the secretion of nitric oxide (NO) by ECs through the activation of the PI3K/Akt pathway and collaborates with TGF-β signaling to induce EndMT (Chang et al., 2011). In addition, disease-focused evidence suggests that the MMP-9/Notch-1 axis participates in EndMT-associated atherosclerotic remodeling. Inhibition of MMP-9 has been reported to attenuate EndMT and improve atherosclerotic pathology by downregulating Notch-1 signaling (He et al., 2024). These findings support a link among Notch activation, matrix degradation, and EndMT-related plaque instability, although additional *in vivo* lineage-resolved studies are needed to define cell-specific Notch functions in plaques.

#### 3.2.5 Wnt/β-catenin signaling pathway

The Wnt pathway is involved in embryonic development, tissue homeostasis, and vascular remodeling. In the canonical Wnt pathway, Wnt ligands bind receptors such as FZD, ROR1/2, and RYK, stabilize β-catenin, and promote its nuclear translocation. Nuclear β-catenin then induces transcription factors such as SNAI1/2, thereby promoting EndMT (Clevers and Nusse, 2012; Akoumianakis et al., 2022). Primary cilia, acting as mechanosensors, can regulate Wnt/β-catenin activity and interact with TGF-β signaling; loss of cilia increases β-catenin nuclear translocation and enhances EndMT. Atherosclerosis-specific evidence supports a disease-relevant role for Wnt signaling. Wnt2 cooperates with TGF-β signaling to promote EndMT and has been identified as a regulatory factor in atherosclerosis development (Zhang et al., 2021). *In vitro*, shRNA-mediated Wnt2 inhibition attenuates Wnt2-driven EndMT, while in Ldlr^−/−^ mice, Wnt2 expression is increased in atherosclerotic plaque regions (Huang et al., 2021). These observations suggest that Wnt/β-catenin signaling contributes to endothelial phenotypic transition and plaque remodeling, although further validation in human plaque samples and lineage-tracing models is still needed.

### 3.3 Transcription factor regulation

Transcription factors integrate extracellular stimuli, metabolic stress, inflammatory signaling, and mechanical cues to regulate endothelial identity and mesenchymal transition. Core EndMT transcription factors include SMADs, SNAI1/2, SLUG, ZEB1/2, TWIST, and ELK1/BACH1 (Lamouille et al., 2014; Kovacic et al., 2019; Xu and Kovacic, 2023). SMAD proteins transmit TGF-β signals and induce pro-mesenchymal gene programs. SNAI1 and SNAI2 suppress endothelial junctional genes, thereby disrupting barrier function. TWIST and ZEB1/2 promote ECM production, cytoskeletal remodeling, and mesenchymal gene expression. ELK1/BACH1 can be activated by shear stress, high glucose, and high-fat diet-associated stimuli, linking metabolic and hemodynamic stress to SNAI-dependent EndMT (Jia et al., 2022; Li et al., 2022).

Inflammatory and metabolic transcription factors also influence EndMT in atherosclerosis-related settings. NF-κB, activated by IL-1β and TNF-α, can upregulate TGF-β1 and TGF-β2, thereby creating a pro-EndMT inflammatory environment (Souilhol et al., 2018). STAT3 has context-dependent effects on EndMT: some studies suggest that STAT3 enhances endothelial migration and SNAI transcription, whereas others indicate that STAT3 may negatively regulate TGF-β1/ALK5/SMAD4 signaling through interaction with SMAD4 (Becerra et al., 2017; Jiao et al., 2020; Dougherty et al., 2023) Sirtuin3 (SIRT3) suppresses EndMT by promoting FOXO3a deacetylation and nuclear translocation (Lin J. R. et al., 2018), whereas FOXO1 under high-glucose conditions may promote EndMT, an effect antagonized by lncRNA DANCR (Wu et al., 2023). C-terminal binding protein 1 (CtBP1), through FOXP1, regulates endothelial inflammation and may contribute to atherosclerosis development (Guo S. et al., 2024).

Several transcriptional regulators have been validated in atherosclerosis-relevant models. HDAC3-related transcriptional programs and α-SMA are increased in ApoE^−/−^ mouse atherosclerotic plaques and in TNF-α/IL-1β-stimulated HUVECs, suggesting a link between inflammatory EndMT and plaque progression (Zeng et al., 2013; Chen et al., 2021). HDAC5 suppresses MEF2-mediated KLF2 transcription, downregulates eNOS, enhances endothelial inflammation, and accelerates atherosclerosis progression (Wang et al., 2010; Kwon et al., 2014). Octamer-binding transcription factor 4 (OCT4) also participates in endothelial homeostasis and plaque remodeling. Shin et al. reported that endothelial cell-specific OCT4 deficiency increased α-SMA expression, reduced mitochondrial Mt-Co1 expression, and promoted EndMT-associated mitochondrial dysfunction through ATP-binding cassette transporter G2 (ABCG2) (Shin et al., 2022). Nuclear Factor, Interleukin 3-Regulated (NFIL3) suppresses MEST promoter activity through H_2_S modification, inhibits EndMT, reduces plaque area, and improves endothelial function (Zhou et al., 2024). Krüppel-like factor 4 (KLF4) has cell-type-specific effects: VSMC KLF4 deficiency may reduce atherosclerotic lesion development, whereas endothelial Klf4 deletion in ApoE^−/−^ mice exacerbates atherosclerosis (Zhou et al., 2012; Shankman et al., 2016). Together with KLF2, endothelial KLF4 is a shear stress-responsive protective transcription factor that maintains endothelial identity and restrains EndMT (He et al., 2022).

Importantly, not all transcription factors associated with atherosclerosis have been directly proven to drive EndMT *in vivo*. When direct EndMT evidence is limited, these regulators should be interpreted as contributors to endothelial activation, EndMT-permissive states, or plaque inflammation rather than definitive EndMT drivers. This distinction helps separate disease-relevant primary evidence from broader mechanistic inference.

### 3.4 Epigenetic regulation

Epigenetic mechanisms, including DNA methylation, histone modifications, chromatin remodeling, and non-coding RNAs, regulate gene expression programs involved in endothelial identity and EndMT. In atherosclerosis, epigenetic regulators can integrate inflammatory, metabolic, and mechanical signals to shape endothelial phenotypic transition (Zhang et al., 2023; Singh A. et al., 2024).

Among epigenetic regulators, HDAC9 provides one of the clearest atherosclerosis-specific examples. Upregulation of HDAC9 in ECs promotes EndMT and contributes to the accumulation of smooth muscle-like and fibroblast-like cells in plaques (Lecce et al., 2021; Ibrahim et al., 2024). These EndMT-associated cells may secrete cytokines and MMPs, intensifying local inflammation and plaque instability. Importantly, HDAC9-related evidence directly links epigenetic regulation, EndMT, and unfavorable plaque phenotype, making it more disease-specific than many general EndMT mechanisms. HDAC3 may also participate in inflammatory EndMT, as HDAC3 inhibition suppresses EndMT in ApoE^−/−^ mice and inflammatory cytokine-stimulated endothelial cells (Zeng et al., 2013; Chen et al., 2021).

Non-coding RNAs, especially lncRNAs and miRNAs, also regulate EndMT by targeting endothelial markers, TGF-β signaling components, and mesenchymal transcriptional programs (Ghosh et al., 2012). MALAT1 enhances TGF-βRII signaling by downregulating miR-145, activating SMAD3 and promoting EndMT (Xiang et al., 2017). Conversely, FGF-induced let-7 miRNA suppresses TGF-β ligands and receptors, thereby inhibiting EndMT (Chen et al., 2012). miR-122 has also been reported to promote atherosclerosis development by regulating NPAS3-mediated EndMT (Wu et al., 2021). These findings suggest that epigenetic and non-coding RNA networks may connect endothelial inflammation, metabolic stress, and EndMT in atherosclerosis. However, because some non-coding RNA studies remain largely cell-model based, future work should validate their plaque-stage specificity, endothelial origin, and therapeutic relevance *in vivo*.

## 4 Therapeutic advances targeting EndMT in atherosclerosis

**FIGURE 3:**
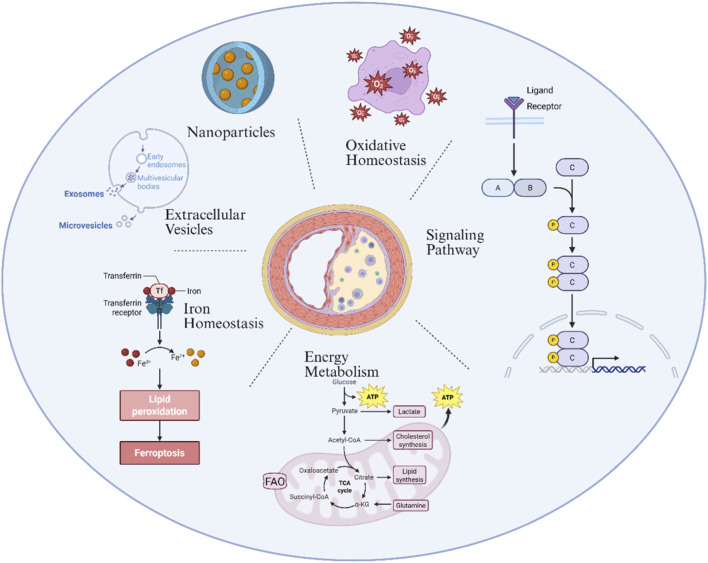
Emerging strategies to curb EndMT in atherosclerosis. Interventions act via: (i) nanoparticle drug carriers; (ii) extracellular vesicles delivering anti-EndMT cargos; (iii) restoration of oxidative and iron balance to block lipid peroxidation/ferroptosis; (iv) metabolic rewiring of glycolysis, FAO and TCA flux; and (v) inhibition of pro-EndMT signalling cascades. Created with BioRender.com.

EndMT is increasingly recognized as a therapeutically modifiable component of endothelial plasticity in atherosclerosis. However, EndMT-targeted therapy should not be understood as complete suppression of all endothelial transition programs. Partial or transient EndMT may contribute to endothelial repair and adaptive extracellular matrix remodeling, whereas sustained inflammatory, osteogenic, fibroblast-like, or matrix-degrading EndMT is more likely to promote plaque vulnerability. Therefore, therapeutic strategies should aim to preserve endothelial identity, prevent or reverse early maladaptive EndMT, and selectively restrain pathogenic EndMT states while maintaining reparative endothelial plasticity (Figure 3; Table 2).

**TABLE 2:** EndMT-directed therapeutic strategies in atherosclerosis: targets, modalities, effects, and translational status.

Target/Class	Intervention	Effect on EndMT	Model/Context	Status/Notes	Ref
Glycolysis/HK1	BDNF → KLF2 axis	KLF2 limits HK1-dependent EndMT; reduces diabetic intimal calcification	Endothelium (diabetic context)	Mechanistic/biologic node	Wang et al. (2022)
Lactate–lactylation (Snail1)	—	Lactate promotes EndMT via Snail1 lactylation after MI; drives fibrosis/remodeling	Post-MI models	Targetable node (block lactate/lactylation)	Fan et al. (2022)
Histone lactylation (H3K18) via p300/ASF1A	—	Accelerates EndMT and atherogenesis	*in vivo*	Targetable node (p300/ASF1A/H3K18lac)	Dong et al. (2024)
PKM2 → lactate → TWIST1 lactylation	—	Triggers EndMT in ischemic tissue	Ischemic flap models	Targetable node (PKM2/lactate/TWIST1)	Xu et al. (2024)
Mitochondrial dysfunction	Frataxin loss	Exacerbates Ang II–induced EndMT; vascular remodeling/fibrosis	Cardiovascular endothelium	Targetable node (mitochondrial QC)	Guo Y. T. et al. (2024)
FA carbon for dNTP synthesis	—	Links FA utilization to EC replication/repair and plaque biology	Endothelium (*in vitro*/*in vivo*)	Targetable node (FAO, nucleotide synthesis)	Schoors et al. (2015)
Enhance FA β-oxidation	Mito-targeted esculetin; metformin	Promotes FAO, delays EC senescence; anti-atherosclerosis relevance	Endothelium/aging models	Preclinical pharmacology	Pulipaka et al. (2024)
Integrated metabolic regulation	Review synthesis	Glycolysis and FA metabolism central to EndMT control	—	Conceptual framework	Xiong et al. (2018)
Iron oxide nanoparticles	SPIO/FeOx NPs	Induce reversible EndMT at non-cytotoxic doses; biosafety signal	Vascular ECs	Nanotoxicology consideration	Wen et al. (2019)
Chemokine signaling	CXCL8 → CXCR2–NF-κB	Drives EndMT and protects from erastin-induced ferroptosis	Endothelium (*in vitro*)	Inflammation–ferroptosis crosstalk	Ji et al. (2023)
TGF-β/ALK5–Smad2/3	Paeonol	Blocks TGF-β2–induced EndMT; reduces fibrosis	HUVEC; *in vivo*	Preclinical small molecule	Yang et al. (2023)
TGF-β production	Pirfenidone	Antifibrotic; approved in IPF; conceptual relevance to EndMT	Clinical (IPF)	Approved drug (repurposing potential)	Nathan et al. (2017)
Notch-1/MMP-9	Morin	Inhibits MMP-9 and Notch-1; weakens EndMT	*in vivo*	Natural flavonoid	He et al. (2024)
STAT3 inhibition	Equisetin	Binds and inhibits STAT3; atheroprotection	*In vivo*	Natural product	Yang et al. (2024)
IL-6 blockade	Anti–IL-6 signaling	Alleviates atherosclerosis in Tet2-deficient clonal hematopoiesis	Preclinical	Cytokine-targeted therapy	Liu W. et al. (2023)
MKL1 → TWIST1	—	Promotes EndMT and fibrosis; potential TF target	Endothelial/fibrosis models	Transcription factor target	Li Z. et al. (2019)
EndMT (epigenetically driven)	Statins	Suppress EndMT via epigenetic mechanisms; reduce remodeling	Preclinical/clinical context	Repurposed class	Liu C. et al. (2023)
FOXP1 → NLRP3 inflammasome	—	FOXP1 suppresses NLRP3 to reduce inflammation	Endothelium; *in vivo*	Transcription factor target	Zhuang et al. (2019)
HDAC9	HDAC9 inhibition	HDAC9 promotes EndMT and unstable plaques; inhibition may stabilize plaques	*in vivo*	Epigenetic target	Lecce et al. (2021)
HDAC9 → miR-17–92 axis	—	HDAC9 promotes angiogenesis by repressing anti-angiogenic miR-17–92	Endothelial cells	Mechanistic link	Kaluza et al. (2013)
miR-483 → CTGF	miR-483 mimic/agonism	Prevents EndMT in Kawasaki disease; anti-remodeling	Clinical tissue; *in vivo*	ncRNA therapeutic concept	He M. et al. (2017)
lncH19/miR-148b-3p/ELF5	Icariin	Regulates H19–miR-148b-3p–ELF5 to inhibit EndMT; protects endothelium	ox-LDL–stimulated HUVECs	Natural compound modulating ncRNA	Liu et al. (2021)
YAP/TAZ (disturbed flow)	Methotrexate	Atheroprotective via inhibition of YAP/TAZ; reduces inflammation/remodeling	Disturbed flow models	Repurposed drug	Liu et al. (2019)
ERK5 → KLF2 → ↓PAK1; ERK5/HDAC5; SIRT1→KLF2	Resveratrol (plus pathway)	ERK5 limits EC migration via KLF2; resveratrol activates ERK5/HDAC5 and SIRT1→KLF2; ↑MnSOD	Endothelial models	Pathway and nutraceutical	Komaravolu et al. (2015), Gan et al. (2016), Gracia-Sancho et al. (2010)
MSC-EVs (general)	MSC-derived EVs	Promote vascular repair/angiogenesis; optimization needed	Multiple models	Biologic platform	Zhu X. L. et al. (2023)
Pharmacologic priming of EVs	Atorvastatin-primed MSC-EVs (↑H19)	Enhanced cardioprotection in AMI	AMI models	Priming strategy	Huang et al. (2020)
Device-based local delivery	Lp-PLA2–triggered exosome stent	Suppresses in-stent restenosis	Preclinical	Responsive stent platform	Zou et al. (2022)
MSC secretome/EVs	Adipose-derived MSC factors	Ameliorate atherosclerosis in Ldlr^−/−^ mice	Ldlr^−/−^ mice	Biologic therapy	Takafuji et al. (2019)
Endothelial barrier stabilization	MSC microvesicles (HGF-mediated)	Stabilize endothelial barrier function	*In vitro*/*in vivo*	Biologic mechanism	Wang et al. (2017)
Donor state risk	Type-2 diabetic MSC-EVs	High-glucose EVs induce EndMT via TGF-β/Smad3 → pro-fibrotic risk	*In vitro*	Quality/safety consideration	Vuong et al. (2024)
Targeted nanocarriers	VCAM-1 shRNA-Smad3 nanocarriers	Mitigate high-glucose/osteogenic factor–induced EndMT in VECs	Valvular EC models	Gene-silencing delivery	Voicu et al. (2024)
Tissue-engineered grafts	Immune-stealth, TGF-β blockade graft	Prevent graft EndMT; improve patency	Preclinical graft models	Bioengineered conduit	Fan et al. (2023)
Cell therapy tracking/modulation	SPIO-labeled EPCs	Inhibit heterotopic ossification; EPC tracking/therapeutic angle	Rat models	Cell + nanoparticle approach	Zhang et al. (2019)
Nanoparticle safety/modulation	Iron oxide nanoparticles	Reversible EndMT at non-cytotoxic doses; consider in design	Vascular ECs	Safety/biological effect	Wen et al. (2019)

### 4.1 Reversibility, prevention, and stage-specific intervention

From a therapeutic perspective, EndMT-targeted strategies can be broadly divided into prevention of EndMT initiation, reversal of partial EndMT, and blockade of maladaptive EndMT progression. Prevention may be most relevant in early atherosclerosis, when disturbed flow, oxidative stress, hyperglycemia, inflammatory cytokines, and lipid stress induce endothelial dysfunction and partial EndMT (Mehta et al., 2021). At this stage, preserving endothelial identity may reduce endothelial permeability, leukocyte adhesion, macrophage recruitment, and early matrix remodeling.

In early-to-intermediate lesions, partial EndMT may remain reversible if pro-EndMT stimuli are removed or pharmacologically counteracted before stable mesenchymal-like programs are established (Zhao et al., 2023; Dong et al., 2024). In advanced plaques, however, the therapeutic goal should shift from preventing lesion formation to stabilizing rupture-prone plaques by selectively limiting inflammatory, osteogenic, or matrix-degrading EndMT. Complete inhibition of EndMT in advanced lesions may be undesirable because partial EndMT-like responses may contribute to reparative extracellular matrix deposition and fibrous cap maintenance (Newman et al., 2021; Tardajos Ayllon et al., 2026). Therefore, optimal clinical application of EndMT-targeted therapy will require disease-stage-specific intervention, plaque-risk stratification, biomarkers of active pathogenic EndMT, and plaque- or endothelium-targeted delivery systems.

### 4.2 Metabolic and mitochondrial targets

Metabolic reprogramming provides a therapeutically relevant layer of EndMT regulation. Glycolytic activation, lactate accumulation, acetyl-CoA metabolism, and lactylation connect vascular metabolic stress to transcriptional EndMT programs. F. Wang et al. demonstrated that KLF2 mediates the inhibitory effect of brain-derived neurotrophic factor on diabetic intimal calcification by limiting HK1-dependent EndMT, highlighting glycolysis as a potential therapeutic target (Wang et al., 2022). Lactate also functions as a signaling effector. After myocardial infarction, lactate promotes EndMT through Snail1 lactylation, contributing to vascular fibrosis and remodeling (Fan et al., 2022). ASF1A-dependent, p300-mediated H3K18 lactylation accelerates EndMT and atherogenesis (Dong et al., 2024). Similarly, PKM2-driven lactate overproduction in ischemic tissues induces EndMT through TWIST1 lactylation, linking metabolic flux to pro-EndMT transcriptional programs (Xu et al., 2024). These findings suggest that targeting glycolytic enzymes, lactate production, lactylation machinery, or related transcriptional programs may be especially relevant in diabetes-associated or hypoxic plaque microenvironments.

Mitochondrial quality control and fatty acid metabolism are also important therapeutic nodes. Loss of the mitochondrial protein frataxin increases susceptibility to angiotensin II-induced EndMT, intensifying vascular remodeling and fibrosis (Guo Y. T. et al., 2024). Endothelial fatty acids provide a carbon source for deoxyribonucleotide synthesis, linking fatty acid utilization to endothelial proliferation, vascular repair, and plaque remodeling (Schoors et al., 2015). Pharmacological enhancement of fatty acid β-oxidation may help maintain endothelial homeostasis. Mitochondria-targeted agents such as esculetin and metformin delay endothelial senescence by promoting β-oxidation, suggesting a potential strategy to mitigate age-related atherosclerosis (Pulipaka et al., 2024). These findings identify mitochondrial quality control and fatty acid metabolism, including fatty acid uptake, β-oxidation, and nucleotide biosynthesis, as tractable targets for modulating EndMT and limiting atherosclerotic progression (Xiong et al., 2018).

Iron overload and ferroptosis-related stress represent additional therapeutic targets. Iron oxide nanoparticles can trigger reversible EndMT at non-cytotoxic concentrations, highlighting nanoparticle exposure as both a potential vascular risk factor and an important biosafety consideration (Wen et al., 2019). CXCL8 induces EndMT through CXCR2–NF-κB activation while protecting endothelial cells from erastin-induced ferroptosis, linking chemokine signaling to EndMT–ferroptosis crosstalk (Ji et al., 2023). Strategies that reduce iron overload, limit ferroptosis, or restore mitochondrial antioxidant capacity may indirectly restrain EndMT-associated endothelial injury, although direct *in vivo* evidence linking iron-dependent EndMT blockade to plaque stabilization remains limited.

### 4.3 Signaling pathway targets

TGF-β/ALK5/SMAD signaling remains one of the central pharmacological entry points for EndMT inhibition. Paeonol reduces vascular fibrosis and improves atherosclerotic pathology by inhibiting the ALK5-SMAD2/3 pathway and blocking TGF-β2-induced EndMT (Yang et al., 2023). Pirfenidone, which suppresses TGF-β production and has been used clinically for antifibrotic therapy, also provides a pharmacological example of targeting TGF-β-related fibrosis (Nathan et al., 2017). ALK5 kinase inhibitors such as LY2157299, EW-7197, and LY3200882 are being evaluated in clinical trials for cancer, fibrosis, and immune-related diseases, suggesting potential translational relevance for TGF-β/ALK5-targeted strategies.

However, TGF-β signaling is highly context dependent in atherosclerosis. While endothelial TGF-β activation may promote EndMT, inflammation, and endothelial barrier disruption, TGF-β signaling in VSMCs may support contractile phenotype maintenance and fibrous cap stability. Therefore, broad systemic blockade of TGF-β may carry risks. Future strategies should emphasize endothelial- or plaque-targeted delivery, pathway modulation rather than complete inhibition, and careful assessment of fibrous cap integrity.

The FGFR1–TGF-β regulatory axis may represent a more refined therapeutic strategy because it restores an endogenous anti-EndMT mechanism rather than broadly suppressing TGF-β signaling. Preservation of endothelial FGFR1 signaling can restrain TGF-β activity, maintain endothelial identity, and limit EndMT-associated plaque remodeling. Therapeutic strategies that prevent FGFR1 degradation, restore FGF/FGFR1 signaling, or enhance downstream endothelial-protective pathways may therefore be particularly attractive for early-to-intermediate atherosclerosis, when endothelial identity loss remains potentially reversible.

Notch and MMP-related pathways also provide therapeutic opportunities. Morin, a flavonoid compound derived from white mulberry, delays atherosclerosis progression by inhibiting MMP-9 activity and downregulating Notch-1 signaling, thereby weakening EndMT (He et al., 2024). However, Notch also regulates vascular development, immune responses, and endothelial homeostasis. Therefore, Notch-targeted strategies will require cell-type-specific modulation and careful safety evaluation.

### 4.4 Transcriptional and epigenetic targets

Several transcriptional regulators have been proposed as therapeutic targets. Equisetin protects against atherosclerosis by inhibiting STAT3 activity, suggesting that STAT3 modulation may have therapeutic value (Yang et al., 2024). IL-6 signaling contributes to atherosclerosis, particularly in the setting of Tet2-deficient clonal hematopoiesis, where IL-6 blockade alleviates disease progression (Liu W. et al., 2023). MKL1 promotes EndMT through TWIST1 activation, highlighting MKL1 as a possible target for limiting EndMT and vascular fibrosis (Li Z. et al., 2019). Statins, beyond their lipid-lowering effects, may suppress EndMT through epigenetic mechanisms and reduce vascular fibrosis and remodeling (Liu C. et al., 2023). Foxp1 inhibits NLRP3 inflammasome activation and reduces inflammation and atherosclerosis, suggesting a potential role in endothelial protection (Zhuang et al., 2019).

Restoring endothelial identity may be safer than broadly blocking mesenchymal transition. Flow-sensitive protective pathways such as KLF2/KLF4, ERK5, SIRT1, and laminar-flow-associated signaling maintain endothelial quiescence, eNOS expression, anti-inflammatory function, and barrier integrity. Methotrexate has been reported to exert atheroprotective effects by modulating YAP/TAZ-dependent mechanotransduction pathways related to vascular inflammation and remodeling (Liu et al., 2019). ERK5 regulates endothelial migration and vascular injury through KLF2-dependent PAK1 modulation (Komaravolu et al., 2015). Resveratrol activates ERK5 signaling and regulates HDAC5 deacetylation, promoting manganese superoxide dismutase expression and reducing oxidative stress-induced vascular damage (Gan et al., 2016). Resveratrol also activates SIRT1, induces KLF2 expression, improves endothelial function, and lowers cardiovascular disease risk (Gracia-Sancho et al., 2010).

Epigenetic regulation is another promising but challenging therapeutic layer. HDAC9 promotes EndMT and contributes to an unfavorable atherosclerotic plaque phenotype; therefore, HDAC9 inhibition may enhance plaque stability by reducing EndMT-associated fibroblast-like and smooth muscle-like cell accumulation, cytokine production, and matrix remodeling (Lecce et al., 2021). HDAC9 also regulates angiogenesis by targeting the antiangiogenic miR-17–92 cluster, indicating that its vascular effects extend beyond EndMT alone (Kaluza et al., 2013). HDAC3 and HDAC5-related pathways may also participate in inflammatory EndMT and endothelial identity loss. However, epigenetic drugs often have broad effects across cell types; long-term safety, immune effects, vascular-bed specificity, and endothelial selectivity must therefore be carefully evaluated before clinical translation.

### 4.5 Non-coding RNA and extracellular vesicle-mediated modulation of EndMT

Non-coding RNAs provide additional opportunities for EndMT modulation because they can regulate endothelial markers, TGF-β receptors, transcription factors, inflammatory signaling, and matrix-remodeling genes. miR-483 targets connective tissue growth factor and prevents EndMT in Kawasaki disease, suggesting a potential approach for vascular inflammation and remodeling (He M. et al., 2017). Icariin inhibits EndMT and protects endothelial function by regulating the lncRNA H19/miR-148b-3p/ELF5 axis, thereby slowing atherosclerosis progression (Liu et al., 2021). Although non-coding RNA-based approaches may offer high specificity, their translation remains limited by delivery efficiency, off-target effects, RNA stability, immune activation, and the need to confirm endothelial origin and plaque-stage specificity *in vivo*.

Extracellular vesicles (EVs) should be considered not only therapeutic delivery platforms but also active biological mediators of EndMT within the vascular microenvironment. EVs released from endothelial cells, macrophages, VSMCs, platelets, adipose tissue, and mesenchymal stromal cells can transfer miRNAs, lncRNAs, proteins, lipids, cytokines, and growth factors to recipient endothelial cells. Depending on their cellular origin and disease context, these cargos may either promote or suppress EndMT by regulating TGF-β/SMAD signaling, inflammatory pathways, oxidative stress, endothelial barrier integrity, and extracellular matrix remodeling.

Mesenchymal stem cell-derived EVs have emerged as promising modulators of vascular repair and regeneration (Zhu Y. et al., 2023). Pharmacological preconditioning can further alter their cargo and biological activity. For instance, atorvastatin increases the cardioprotective activity of MSC-EVs by upregulating lncRNA H19 (Huang et al., 2020). In atherosclerosis, adipose-derived MSC secretome and EVs alleviate disease progression in Ldlr^−/−^ mice, demonstrating anti-inflammatory and reparative effects *in vivo* (Takafuji et al., 2019). MSC-derived microvesicles also stabilize endothelial barrier function partly through hepatocyte growth factor-dependent signaling (Wang et al., 2017). Conversely, the physiological state of source cells may convert EVs into pro-EndMT mediators. EVs derived from type 2 diabetic MSCs cultured under high-glucose conditions can trigger EndMT through activation of the TGF-β/SMAD3 pathway, linking diabetic metabolic stress to pro-fibrotic endothelial transition (Vuong et al., 2024). These findings indicate that EV-associated cargos, including lncRNA H19, hepatocyte growth factor-related protein signaling, TGF-β/SMAD-activating factors, and potentially disease-conditioned miRNAs or inflammatory proteins, can actively shape endothelial phenotype rather than merely serve as passive delivery vehicles.

### 4.6 Targeted delivery platforms and translational challenges

Targeted delivery platforms may help overcome one of the major barriers in EndMT-directed therapy: the lack of pathway specificity. TGF-β/SMAD, Notch, Wnt, STAT3, NF-κB, HDACs, metabolic enzymes, and mechanotransduction pathways regulate multiple vascular and immune cell types. Systemic intervention may therefore produce off-target effects or impair reparative endothelial and VSMC functions. Endothelium-targeted nanocarriers, lesion-responsive vascular stents, EV-based delivery, and tissue-engineered vascular grafts may improve spatial precision and reduce systemic toxicity.

Nanocarriers targeting VCAM-1 have shown promise in mitigating EndMT triggered by high glucose and osteogenic stimuli in valvular endothelial cells (Voicu et al., 2024). Delivery of shRNA-SMAD3 using such targeted platforms may offer a strategy for suppressing TGF-β/SMAD-driven EndMT in metabolically stressed vascular environments. Tissue-engineered vascular grafts designed to mimic immune stealth and block TGF-β signaling have demonstrated improved patency and integration by reducing EndMT-associated graft failure (Fan et al., 2023). Lesion-responsive exosome-loaded vascular stents that release therapeutic cargos in response to Lp-PLA_2_ have also been shown to suppress in-stent restenosis in preclinical studies (Zou et al., 2022).

However, not all nanoparticle effects are protective. Iron oxide nanoparticles can induce reversible EndMT in vascular endothelial cells at non-cytotoxic concentrations (Wen et al., 2019), highlighting the need to evaluate EndMT as both a therapeutic endpoint and a potential vascular safety concern in nanomedicine. SPIO nanoparticles have also been used to label endothelial progenitor cells and modulate vascular regeneration-related processes (Zhang et al., 2019), but their long-term effects on endothelial identity, EndMT reversibility, and plaque stability require further investigation.

Several translational challenges must be addressed before EndMT-targeted interventions can be clinically applied. First, there are no clinically validated EndMT-specific biomarkers, making it difficult to identify patients with active pathogenic EndMT or monitor therapeutic response. Second, EndMT is heterogeneous across disease stages, plaque regions, and vascular beds, so one therapeutic strategy may not apply equally to coronary, carotid, aortic, or peripheral arteries. Third, many preclinical models do not fully reproduce human plaque rupture, limiting assessment of plaque-stabilizing efficacy. Fourth, therapies must distinguish maladaptive inflammatory EndMT from reparative endothelial plasticity. Future clinical translation will therefore require composite biomarkers, plaque-risk stratification, vascular-bed-specific validation, and targeted delivery systems capable of selectively modulating pathogenic EndMT within plaques.

## 5 Conclusions and perspectives

EndMT has emerged as an important dimension of endothelial plasticity in atherosclerosis, linking endothelial injury to inflammation, matrix remodeling, calcification, and plaque destabilization. However, EndMT should not be interpreted as a uniform, irreversible, or intrinsically detrimental process. Current evidence instead supports a spectrum model in which endothelial cells acquire partial, intermediate, or advanced mesenchymal-like states depending on the local hemodynamic, metabolic, inflammatory, and spatial context. Within this framework, transient or partial EndMT may contribute to endothelial adaptation, wound repair, extracellular matrix deposition, and fibrous cap remodeling, whereas sustained inflammatory, osteogenic, or matrix-degrading EndMT may aggravate barrier dysfunction, leukocyte recruitment, collagen disorganization, cap weakening, and plaque vulnerability. This conceptual shift is important because it moves the field beyond the simplistic goal of “blocking EndMT” toward a more refined strategy of distinguishing pathogenic, reparative, reversible, and therapeutically actionable EndMT states.

Despite substantial mechanistic progress, several barriers continue to limit clinical translation. The first is the lack of a definitive EndMT marker. Commonly used mesenchymal or fibroblast-like markers, including α-SMA, FSP1/S100A4, COL1A1, fibronectin, and FAP, are not specific to endothelial-derived cells and may also be expressed by VSMCs, fibroblasts, pericytes, or inflammatory stromal populations. Therefore, EndMT identification cannot rely on marker co-expression alone. More robust evidence requires the integration of endothelial lineage tracing or fate mapping, endothelial–mesenchymal marker combinations, single-cell transcriptomic signatures, spatial localization, and functional validation. The second challenge is biological heterogeneity. EndMT programs may differ across disease stages, plaque regions, vascular beds, and metabolic backgrounds. EndMT-like states in coronary, carotid, aortic, or peripheral arteries may not be directly interchangeable because these vascular territories differ in shear stress profiles, endothelial identity, plaque architecture, immune composition, and extracellular matrix organization. The third challenge is model fidelity. Most experimental systems do not fully reproduce the chronicity and complexity of human plaque evolution, particularly the coexistence of disturbed flow, lipid accumulation, metabolic disease, immune-cell crosstalk, intraplaque neovascularization, calcification, and spontaneous rupture (He L. et al., 2017; Hall et al., 2024). These limitations should be considered when extrapolating preclinical EndMT findings to human disease.

Future studies should therefore prioritize models and technologies that preserve the spatial, mechanical, and cellular complexity of atherosclerotic plaques. Human-derived three-dimensional vascular co-cultures, plaque-mimetic matrices, organ-on-chip or vessel-on-chip platforms, and flow-controlled endothelial systems may provide more physiologically relevant settings for studying how disturbed shear stress, oxidized lipids, inflammatory cytokines, oxidative stress, and diabetic metabolic cues converge to induce EndMT. Humanized models and large-animal models may further improve the evaluation of fibrous cap remodeling, lesion architecture, immune context, and plaque vulnerability. Importantly, these systems should not only be used to induce EndMT, but also to determine whether specific EndMT states can be prevented, reversed, or redirected toward reparative phenotypes.

Equally important is the development of state-resolved and spatially resolved EndMT maps. Single-cell RNA sequencing has begun to reveal endothelial heterogeneity and EndMT-like transcriptional states within plaques, whereas spatial transcriptomics can assign these states to biologically distinct niches, including the luminal endothelium, plaque shoulder, fibrous cap, neovessels, necrotic core border, and calcified regions. When combined with endothelial lineage tracing, genetic barcoding, multi-omics, and functional assays, these approaches may help distinguish true EndMT-derived cells from VSMC-, fibroblast-, or pericyte-derived mesenchymal-like populations. They may also clarify whether EndMT-derived cells undergo clonal expansion, how they interact with macrophages and VSMCs, and which transitional states are reversible, persistent, protective, or harmful.

Therapeutically, the most promising strategies are likely to be those that are stage specific and cell-type selective. In early-to-intermediate atherosclerosis, preserving endothelial identity and preventing EndMT initiation may reduce endothelial permeability, leukocyte adhesion, macrophage recruitment, and early matrix remodeling. In more advanced plaques, however, the therapeutic objective should shift from preventing lesion formation to stabilizing rupture-prone plaques by selectively limiting inflammatory, osteogenic, and matrix-degrading EndMT programs. Complete suppression of EndMT may be undesirable, because certain partial EndMT-like responses may contribute to reparative matrix deposition and fibrous cap maintenance. Thus, future interventions should aim to restrain maladaptive EndMT while preserving endothelial repair, adaptive matrix remodeling, physiological angiogenesis, immune balance, and VSMC-mediated fibrous cap integrity.

Several EndMT-related pathways deserve further investigation, including the FGFR1–TGF-β axis, TGF-β/ALK5/SMAD signaling, disturbed-flow mechanotransduction, glycolysis–lactate–lactylation pathways, mitochondrial and fatty acid oxidation programs, oxidative stress and ferroptosis-related mechanisms, HDAC9-mediated epigenetic regulation, selected non-coding RNAs, and endothelial identity regulators such as KLF2/KLF4, ERK5, SIRT1, and eNOS. Nevertheless, many of these pathways are shared by endothelial cells, VSMCs, macrophages, fibroblast-like cells, and immune populations. Broad systemic inhibition may therefore produce unintended effects, including impaired endothelial repair, weakened fibrous cap maintenance, altered immune responses, or disrupted physiological vascular remodeling. Endothelium-targeted, plaque-targeted, or stimulus-responsive delivery systems may help improve therapeutic specificity and reduce off-target toxicity, as illustrated by inflammatory endothelium-targeted and enzyme-responsive nanoparticle platforms that enhance lesion accumulation and suppress plaque inflammation in experimental atherosclerosis (Fang et al., 2022).

From a clinical perspective, patients with diabetes may represent a particularly relevant population for EndMT-targeted intervention, because hyperglycemia, oxidative stress, chronic inflammation, metabolic reprogramming, and disease-conditioned extracellular vesicles can converge to promote pathogenic EndMT and accelerated plaque remodeling (Khan et al., 2026). However, clinical translation will require reliable biomarkers capable of identifying active, pathogenic EndMT rather than nonspecific endothelial injury or stromal activation. Such biomarkers will probably need to combine circulating endothelial injury markers, inflammatory mediators, extracellular vesicle cargos, imaging features, and tissue- or spatial-omics signatures. These composite frameworks could help enrich trial populations, monitor target engagement, define therapeutic windows, and evaluate whether EndMT-directed interventions improve plaque stability rather than merely altering molecular markers.

In summary, EndMT provides a valuable lens through which to understand the dynamic interaction between endothelial injury, inflammation, matrix remodeling, and plaque vulnerability. The next stage of the field should move from cataloguing EndMT-associated pathways toward defining EndMT states with spatial, temporal, and functional precision. When integrated with established lipid-lowering, antithrombotic, metabolic, and anti-inflammatory therapies, selective modulation of maladaptive EndMT may offer a complementary strategy to slow atherosclerosis progression, enhance plaque stability, and reduce cardiovascular risk.

## Glossary

EndMT: Endothelial-to-mesenchymal transition
ECs: Endothelial cells
MMP: Matrix metalloproteinase
ECM: Extracellular matrix
VSMCs: Vascular smooth muscle cells
BMP: Bone morphogenetic protein
TGF-β: Transforming growth factor-β
FGF: Fibroblast growth factor
TβRI: TGF-β receptor I
NO: Nitric oxide
CD31: Cluster of Differentiation 31
α-SMA: α-Smooth Muscle Actin
vWF: von Willebrand Factor
eNOS: Endothelial Nitric Oxide Synthase
ZO1/2: Zonula Occludens 1/2
FSP1: Fibroblast-Specific Protein 1
COL1A1: Collagen Type I Alpha 1 Chain
ZEB1/2: Zinc Finger E-box Binding Homeobox 1/2
TWIST: Twist Family BHLH Transcription Factor
FAP: Fibroblast Activation Protein
FN1: Fibronectin 1
Ac-CoA: Acetyl-Coenzyme A
ACLY: ATP-citrate lyase
PDHC: Pyruvate dehydrogenase complex
ACSS1: Acetyl-CoA synthetase short-chain family member 1
PDK4: Pyruvate dehydrogenase kinase 4
FAO: Fatty acid oxidation
CPT1A: Carnitine palmitoyltransferase 1A
ROS: Reactive oxygen species
BVR: Biliverdin reductase
LPO: lipid peroxidation
